# A rare tropical storm event drives partial nursery evacuation by juvenile white sharks, followed by rapid aggregation reformation

**DOI:** 10.1186/s40462-026-00642-0

**Published:** 2026-03-04

**Authors:** Jack T. Elstner, Emily Spurgeon, Patrick Rex, Elizabeth Jahn, Zachariah Merson, Whitney Jones, Lauren Faulkner, James Anderson, Ryan Logan, Wave Moretto, Theodora Mautz, Rilee Sanders, Max Titcomb, Gabriel Gekas, Christopher G. Lowe, Brice X. Semmens

**Affiliations:** 1 Scripps Institution of Oceanography, University of California San Diego, La Jolla, Biological Grade, CA 8750 USA; 2 Department of Biological Sciences, California State University Long Beach, 1250 Bellflower Blvd, Long Beach, CA 90840 USA; 3 Atlantic White Shark Conservancy, 235 Orleans Rd, North Chatham, MA 02633 USA

**Keywords:** White sharks, Shark nurseries, Tropical storms, Bayesian methods, State space models

## Abstract

Extreme weather events, such as tropical storms, can pose profound disruption to nearshore marine environments. Although coastal ecosystems are particularly vulnerable to storm impacts, research describing the response of marine taxa to extreme weather remains limited, especially for highly mobile marine predators. In this study, we use acoustic telemetry to investigate the behavioral responses of juvenile white sharks (*Carcharodon carcharias*) at a Southern California nursery aggregation site to Tropical Storm Hilary in 2023. To aid in these efforts, we developed a novel occupancy modeling approach to statistically account and correct for temporal variations in acoustic receiver performance during disruptive storm conditions. Using detection data from synchronization transmitters, we first estimated the effects of key environmental factors on transmitter detection efficiency, with ambient noise, receiver tilt, and the density of animal transmitters present inside the receiver array emerging as particularly influential predictors. We then leveraged these estimated effects to inform a Bayesian state space model designed to investigate nursery occupancy dynamics, including environmental drivers of nursery emigration. Our results provide evidence of partial nursery evacuation, with over half of tagged sharks temporarily leaving monitored nearshore habitats in response to peak storm conditions. However, most emigrations were short-lived, with all but one shark returning to the aggregation site within three weeks of the storm. Evacuations from nursery habitats were best predicted by falling sea surface temperatures, although increased wave height, declining barometric pressure, and drops in salinity may have served as important secondary flight cues. Our efforts provide a rare opportunity to document the storm response behaviors of a recovering top predator, while also presenting tangible analytical solutions to commonly encountered technical challenges in the field of acoustic telemetry.

**Supplementary information:**

The online version contains supplementary material available at 10.1186/s40462-026-00642-0.

## Introduction

Understanding how animals respond to changing environmental conditions is a foundational aspect of ecology and paramount to effective species conservation and management [1, 2]. In general, organisms select for habitats that maximize growth, survival, and reproductive success, while minimizing threats posed by biophysical and ecological stressors [3]. However, when environmental conditions deteriorate past physiological tolerance limits, species are expected to adaptively respond [4–7]. Such strategies may include either shifting distributions towards more favorable habitats [8, 9] or adopting behaviors that enhance fitness during suboptimal conditions. [10, 11]. To date, most ecological studies examining the impacts of environmental variability on animal movement and behavior have focused on environmental changes that are relatively gradual or predictable in nature, such as tides, seasonal temperature cycles, changes in day length, or long-term climate variability [12–14]. In contrast, relatively few studies have documented how animals respond to environmental disturbances that are stochastic, abrupt, or extreme [7, 9, 15].

Hurricanes and tropical storms are extreme weather events that pose profound risk to coastal communities that lie in their path [16, 17]. These events often occur infrequently relative to the life history of species they impact, yet still have the capacity to drastically alter survivorship of both individuals and populations [9]. Coastal marine ecosystems are particularly vulnerable to catastrophic storms [18]. The effects of turbulent conditions caused by high winds and large swell are amplified in shallow waters, often resulting in the physical alteration and destruction of critical habitats [19, 20]. Wave mixing and wind-induced upwelling can also drive acute changes to the thermal environment [21], possibly pushing temperature conditions beyond species’ thermal tolerance limits. Rapid changes to coastal hydrology, such as heavy precipitation, increased river discharge, and storm runoff, can impact local biogeochemical conditions (e.g., salinity, dissolved oxygen, turbidity) and modify the delivery of nutrients, sediment, and contaminants to nearshore ecosystems [22–24]. For animals occupying impacted habitats, such environmental shifts can necessitate increased energy expenditure, limit foraging opportunities, or even cause mortality [9, 25]. With anthropogenic climate change, tropical storms and the ecosystem disturbances they cause will likely become stronger and more frequent [26, 27]. Therefore, there is an urgent need to understand how marine taxa respond to, and recover from extreme weather events to better evaluate their vulnerability and adaptive capacity in a changing ocean.

During extreme weather events, some coastal species have been shown to modify their spatial ecology in ways that increase chances of survival [9, 15, 18, 28]. Possible behavioral responses include emigration or evacuation behavior, where individuals respond to proximal environmental cues by leaving nearshore or estuarine habitats for calmer, deeper, offshore waters [29]. Falling barometric pressure has been identified as one such emigration cue for several species. For instance, the departure of sea snakes from littoral habitats in Taiwan was observed to be highly correlated with acute barometric pressure changes prior to the arrival of a strong typhoon [30]. Similar responses have been observed in juvenile bull sharks (*Carcharhinus leucas*) and blacktip sharks (*Carcharhinus limbatus*), which emigrated from nursery habitats in coastal bays and estuaries prior to the landfall of major Atlantic hurricanes [9, 15]. Flight behavior from home range habitats has also been documented in teleost fishes such as gray triggerfish (*Balistes caprisus*), striped bass (*Morone saxtilis*), and black sea bass (*Centropristis striata*) [5, 18, 28]. These responses were thought to be primarily driven not by changes in barometric pressure, but instead by increased wave action, high river discharge, rapid drops in salinity, and thermal changes. Still other studies document highly variable storm responses within and between species, where individuals and taxa vary in their propensity to stay or leave [4, 5, 7]. Ultimately, understanding how marine species respond to storm-induced stressors and quantifying their relative sensitivity to disturbance enables the implementation of management strategies that are proactive, adaptive, and resilient to future change.

Acoustic telemetry has become a standard tool for studying the movements, residency, and activity patterns of aquatic species [31–33]. Networks of underwater acoustic receivers, strategically deployed across habitats of interest, provide a means to continuously monitor tagged animals in their natural habitats as environmental conditions change. In theory, acoustic tracking represents a promising opportunity to examine storm response behavior in marine taxa, due to the ability of receiver arrays to continuously monitor animal movements before, during, and after disturbance events. However, past research in this area has been largely opportunistic and limited by several technical challenges. First, the availability of high-quality acoustic telemetry data is rare for periods encompassing hurricanes and tropical storms. Not only must acoustic receivers be serendipitously deployed in the direct path of inherently unpredictable storm systems, they must also withstand their power and remain on station [34]. To prevent destruction of gear and data loss, researchers often recover receivers prior to the storm’s arrival, resulting in critical data gaps. In addition, several environmental factors during storms, particularly wind, wave action, and ambient noise, have been demonstrated to degrade the performance of acoustic receivers and reduce detection probability of acoustic signal transmissions [35, 36]. Such changes to receiver performance, when not carefully considered, have the potential to bias study conclusions [34, 37–39]. However, while many studies acknowledge these impacts and limitations, few explicitly account and correct for storm-driven variations in detection efficiency and seldom incorporate observational uncertainty into models of animal movement [40]. Thus, developing quantitative approaches to address these issues represents a critical next step.

In August 2023, Tropical Storm Hilary brought destructive winds, historic rainfall, and intense flooding to many regions of Mexico and the southwestern United States. This rare cyclone caused the National Hurricane Center to release its first ever Tropical Storm Warning for Southern California and resulted in over $900 million in damages in the United States alone [41]. Tropical Storm Hilary reached peak intensity as a Category 4 Hurricane on 18 August 2023 but was downgraded to a tropical storm prior to making landfall along the coast of northwestern Baja California Sur (345 km southeast of San Diego, CA). Although far less extreme than originally forecasted, coastal communities from San Diego to Los Angeles experienced brief, yet pronounced gale-force winds that resulted from the remnants of Tropical Storm Hilary and a newly formed non-tropical low-pressure system. Many locations also set daily and/or monthly rainfall records, causing significant flash and riverine flooding throughout the region [41].

Here, we use acoustic telemetry and novel occupancy modeling approaches to quantify the behavioral responses of juvenile white sharks (*Carcharodon carcharias*; JWS) to Tropical Storm Hilary. This project stemmed directly from broader efforts to understand fine-scale JWS movements within coastal nursery aggregations throughout the Southern California Bight [42–44], which represents a prominent nursery region for recovering white shark populations in the Northeast Pacific [14, 45–48]. Because young-of-year and juveniles represent the most vulnerable life stages for white sharks, disruptions to nursery habitats have the potential to produce long-term, population-level impacts and hinder ongoing recovery efforts. Therefore, the primary aim of this study was to understand how JWS responded to Tropical Storm Hilary, as well as identify environmental stressors that drove changes to JWS nursery habitat use. To account for temporal variations in receiver performance over our study period, we first analyzed acoustic detection data from synchronization transmitters. This allowed us to probabilistically estimate reductions in transmitter detection efficiency during storm conditions and identify key factors influencing receiver performance. We then integrated this information into probabilistic models of shark movement designed to quantify storm-driven changes in nursery occupancy. Through these efforts, we provide a unique lens into understanding how marine taxa respond to environmental disturbance events, which may ultimately inform vulnerability assessments of individuals, populations, and ecosystems. Moreover, our efforts present tangible analytical solutions to commonly encountered technical challenges in the field of acoustic telemetry, which are routinely reported but seldom corrected for.

## Methods

### Shark tracking

**Fig. 1:**
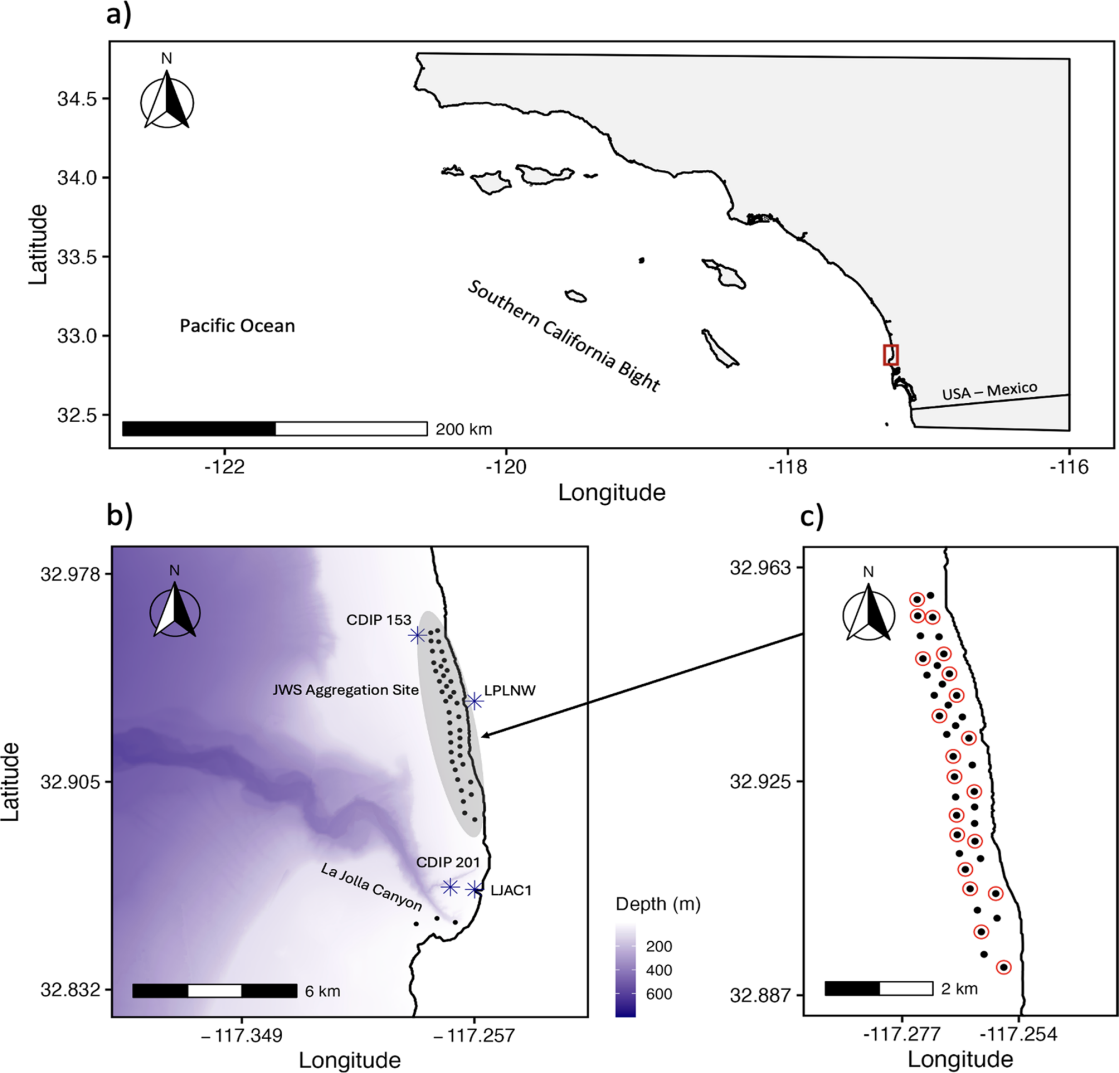
**a**) Location of study site (red rectangle) in San Diego, CA, USA. **b**) Map of acoustic receiver positions (black dots) and environmental monitoring stations (blue asterisks) overlayed onto a regional digital elevation model (1930 – 2014 USGS CoNED Topobathy DEM, 2016: Southern Coast of CA & Channel Islands) [49]. Core juvenile white shark nursery habitats are shown in gray. **c**) Map of fine-scale receiver array. Red circles indicate receivers equipped with internal synchronization transmitters

We used acoustic telemetry to monitor tagged JWS throughout shallow coastal habitats offshore of Torrey Pines and Del Mar, California (Fig. 1), an area that has recently been identified as a prominent JWS nursery aggregation site [45, 47, 50]. We fit sharks with external InnovaSea coded acoustic transmitters using methods described in Anderson et al., 2021 and Spurgeon et al. 2022. Sharks were fit with three distinct types of transmitters: V16P-6x-BLU-2-0204 m transmitters (nominal delay = 150–250 sec, 10-year battery life), V13AP-1x-BLU-3 transmitters (nominal delay = 150–250 sec, 622-day battery life), and V16TP-6x-BLU-2-0204 m transmitters (nominal delay = 150–250 sec, 10-year battery life). All transmitters were also equipped with pressure sensors (204 m depth rating and 0.9 m depth resolution) that reported animal depth at the time of signal transmission. All sharks included in this analysis were tagged between 9 June 2022 and 13 July 2023 (Table 1). Fieldwork was conducted in full compliance with necessary permitting requirements (State of California Department of Fish and Wildlife permit S-200570001-20105–001) and approved under the California State University Long Beach Institutional Animal Care and Use Committee (IACUC); protocol 2019–003-FLD.

**Table 1:** Tagging summary of all sharks

Shark ID	Tag Family	Tagging Date (mm/dd/yy)	Sex	Length at Tagging (cm)	Estimated Shark Age During Storm (yrs)	Total Detections
2022–24	V16TP-6x-BLU-2-0204 m	06/09/22	F	213.4	3.3	28,940
2022–32	V16P-6x-BLU-2-0204 m	06/23/22	M	243.8	4.3	3
2022–49	V16TP-6x-BLU-2-0204 m	08/09/22	F	213.4	3.2	1,449
2022–53	V16P-6x-BLU-2-0204 m	08/12/22	F	213.4	3.1	29,474
2022–55	V16P-6x-BLU-2-0204 m	08/12/22	F	243.8	4.1	20,433
2022–56	V16P-6x-BLU-2-0204 m	08/12/22	F	182.9	2.2	29,310
2022–57	V16P-6x-BLU-2-0204 m	08/12/22	F	213.4	3.1	28,020
2022–63	V16P-6x-BLU-2-0204 m	09/02/22	F	243.8	4.1	31,865
2022–78	V16P-6x-BLU-2-0204 m	09/26/22	M	213.4	3	29,323
2022–82	V16P-6x-BLU-2-0204 m	10/10/22	M	213.4	3	19,441
2022–83	V16P-6x-BLU-2-0204 m	10/10/22	M	213.4	3	30,085
2023–01	V13AP-1x-BLU-3	06/09/23	M	213.36	2.3	25,837
2023–02	V16P-6x-BLU-2-0204 m	06/10/23	F	213.36	2.3	24,392
2023–05	V13AP-1x-BLU-3	07/07/23	F	198.1	1.8	25,093
2023–06	V13AP-1x-BLU-3	07/07/23	F	213.4	2.2	20,828
2023–08	V16P-6x-BLU-2-0204 m	07/13/23	F	243.8	3.2	28,989
2023–09	V16P-6x-BLU-2-0204 m	07/13/23	M	182.9	1.3	33,264
2023–10	V16P-6x-BLU-2-0204 m	07/13/23	M	198.1	1.8	25,239
2023–12	V16P-6x-BLU-2-0204 m	07/13/23	M	213.4	2.2	16,265
2023–13	V16P-6x-BLU-2-0204 m	07/13/23	M	198.1	1.8	32,918
2023–14	V16P-6x-BLU-2-0204 m	07/13/23	F	243.8	3.2	11,169
2023–15	V16P-6x-BLU-2-0204 m	07/13/23	F	198.1	1.8	32,263

We monitored tagged sharks using two distinct acoustic receiver arrays (Fig. 1). The first was a gridded fine-scale array of 40 omni-directional VR2W (*n* = 20) and VR2Tx (*n* = 20) acoustic receivers deployed throughout the JWS aggregation site. These receivers were moored in shallow coastal waters ~1 m above the seafloor in depths ranging from 5 to 15 m. Receiver stations were spaced approximately 300–500 m apart and provided coverage of ~ 10 km^2^ of nearshore habitat. The geometric configuration of our fine-scale array was informed by previous studies in similar environments [42, 44, 45] and selected to maximize detection probability of tagged sharks within core JWS nursery habitats. Randomized transmitter pulse intervals between 150 and 250 sec supported at least hourly detectability of up to 25 tagged animals in the same location at the same time, while reducing the probability of signal code collisions.

Although the analysis presented here leverages only raw receiver detection data, we designed our fine-scale array to function as a Vemco Positioning System (VPS). VPS arrays use overlapping receiver detection ranges, signal time-difference-of arrival (TDOA), and ultimately hyperbolic tri-lateration to generate fine-scale geolocations of tagged animals [51, 52]. This approach allows for semi-continuous, long-term animal tracking at increasingly fine spatial and temporal scales, and has emerged as a widely adopted tool in fisheries science [12, 53–55]. To ensure accurate TDOA calculations, VPS arrays require all receivers to possess internal clocks that are highly synchronized with respect to time. However, because receivers are autonomous devices that are subject to clock drift, stationary synchronization transmitters (hereafter ‘sync tags’) are used to correct for clock skew between neighboring pairs of receivers [52]. In this study, our VPS array included 20 active sync tags, which were internal components of all deployed VR2Tx receivers. Therefore, in addition to recording detections of tagged sharks, receivers also continuously logged detections for sync tag transmissions, providing a unique metric for quantifying temporal variations in acoustic receiver performance. All sync tags were set to “medium” power output with a nominal transmission delay of 540–660 sec.

In addition to our primary fine-scale array, we also leveraged detection data from a broader network of VR2W acoustic receivers deployed throughout coastal habitats in San Diego. Particularly valuable to this study was the La Jolla Receiver Array, which is maintained by the Scripps Institution of Oceanography at UC San Diego. This second array provided coverage of habitats in La Jolla Cove, located approximately 7 km south of the aggregation site’s core area.

### Environmental data

Our study site was located proximal to a diverse suite of meteorological, oceanographic, and hydrologic monitoring stations that provided hourly coverage of changing environmental conditions during Tropical Storm Hilary. In this analysis, we examined the response of JWS to six environmental storm predictors: wind speed, significant wave height, sea surface temperature, barometric pressure, salinity, and turbidity (Table 2). All environmental data were measured at an hourly sampling resolution and were collected within 5 km of the aggregation site’s core area, making them broadly representative of physical and biogeochemical conditions experienced in nearshore habitats.

**Table 2:** Description of environmental parameters and receiver metadata used in models of receiver performance and shark movement

Parameter	Abbreviation	Data Source	Description
Peak Wind Speed	*WIND*	National Oceanic and Atmospheric Administration (NOAA)	Hourly records of peak wind speed (*m/s*) recorded at NOAA meteorological station LJAC1 (32.86703, −117.25714)
Significant Wave Height	*WAVES*	Coastal Data Information Program (CDIP)	Hourly records of significant wave height (*m*) recorded at CDIP Buoy 153 (32.95654, −117.27956)
Sea Surface Temperature	*SST*	Coastal Data Information Program (CDIP)	Hourly records of sea surface temperature (*°C*) recorded at CDIP Buoy 201 (32.86785, −117.26667)
Barometric Pressure	*BARO*	National Oceanic and Atmospheric Administration (NOAA)	Hourly records of barometric pressure (*hPa*) recorded at NOAA meteorological station LJAC1 (32.86703, −117.25714)
Salinity	*SAL*	Los Peñasquitos Lagoon Foundation	Hourly records of salinity (*psu*) recorded at the mouth of the Los Peñasquitos Lagoon (Monitoring Station LPLNW: 32.93333, −117.25721)
Turbidity	*TURB*	Los Peñasquitos Lagoon Foundation	Hourly records of turbidity (*FNU*) recorded at the mouth of the Los Peñasquitos Lagoon (Monitoring Station LPLNW: 32.93333, −117.25721)
Ambient Noise	*NOISE*	Deployed VR2Tx Acoustic Receivers	Hourly records of ambient noise (*mV*) averaged across all 20 deployed VR2Tx acoustic receivers
Receiver Tilt	*TILT*	Deployed VR2Tx Acoustic Receivers	Hourly records of receiver tilt (deviation from vertical, measured in *degrees*) averaged across all 20 deployed VR2Tx acoustic receivers

In addition to the environmental covariates described above, we also obtained hourly records of ambient noise and receiver tilt (e.g., deviation from vertical, in degrees) from each of the 20 VR2Tx receivers deployed throughout the array. We averaged measured values across all VR2Tx receivers to build a time series of hourly means for each covariate.

### Statistical analysis

The primary analytical objective of this study was to estimate hourly presence or absence of tagged sharks within our array and relate shifts in nursery occupancy to storm-induced environmental changes. However, because the same environmental factors that potentially initiated emigration behavior also influence acoustic detection probability, we needed to develop methods to statistically partition changes in true latent states of shark behavior from changes in the ability of receivers to detect tagged animals. To accomplish this, we first analyzed detection data from sync tags, which were continuously deployed at fixed locations within the array, to estimate environmental effects on transmitter detection efficiency. We then leveraged these estimated effects to inform the fitting of a Bayesian state space model of shark nursery occupancy.

Prior to fitting models, we first filtered our acoustic detection database to include only JWS and sync tag detections from 01 August to 10 September 2023. This time period encompassed the arrival of Tropical Storm Hilary on 20 August 2023, enabling us to quantify shark movement patterns and acoustic receiver performance before, during, and after the storm. We also inferred the approximate age of each shark at the time of the storm using estimates of shark size at the time of tagging and von Bertalanffy growth parameter values provided by Cailliet et al., 1985 [56]. Next, for both shark and sync tag detections, we transformed raw detection data into an hourly detection matrix (e.g., detection = 1, non-detection = 0) across all transmitters, receivers, and time steps.

### Array performance

We used a Bayesian logistic regression and Markov Chain Monte Carlo (MCMC) simulations to model hourly sync tag detections recorded on neighboring receivers. We modeled the binary hourly detection outcome $y_{i,j,t}^{sync}$ for sync tag *i* on receiver *j* at time *t* as a Bernoulli distributed response variable, with a detection probability of $p_{i,j,t}^{sync}$.

$$y_{i,j,t}^{sync} \sim\ Bernoulli\left( {p_{i,j,t}^{sync}} \right)$$

We then used a logit link function to quantify the relative effects of various static and dynamic environmental predictors on sync tag detection probabilities. Storm predictors, which were variable in time, included wind speed (*WIND*_*t*_), significant wave height (*WAVES*_*t*_), ambient noise (*NOISE*_*t*_), and receiver tilt (*TILT*_*t*_). These covariates were selected based on factors demonstrated to impact receiver performance in other acoustic tracking studies and believed to be putatively important in our study system. [34–36, 38, 57, 58]. Prior to model fitting, we assessed for potential collinearity between environmental predictors, which was determined to be minimal. We also examined the influence of sync tag - receiver distance (*DIST*_*i,j*_), which was log-transformed, and receiver depth (*DEPTH*_*j*_) to explain changes in detection efficiency stemming from array geometry and receiver deployment depth. Lastly, we included the number of sharks detected in the array per hour (*SHARKS*_*t*_) as a covariate to assess whether variable animal transmitter densities affected sync tag detection probabilities via signal code collisions.

$$\begin{aligned}logit\left( {p_{i,j,t}^{sync}} \right)&= \alpha _{i,j}^{sync} + \beta _{dist}^{sync}logDIS{T_{i,j}} \cr&\quad+ \beta _{depth}^{sync}DEPT{H_j}+ \beta _{wind}^{sync}WIN{D_t} \cr&\quad+ \beta _{waves}^{sync}WAVE{S_{t }} + \beta _{noise}^{sync}NOIS{E_t} \cr&\quad+ \beta _{tilt}^{sync}TIL{T_t} + \beta _{sharks}^{sync}SHARK{S_t}\end{aligned}$$

In our model formulation, we included a unique intercept for each sync tag - receiver pair ($\alpha _{i,j}^{sync}$), which allowed us to estimate baseline pairwise detection levels, which were further modified by static and time-varying environmental predictors. We specified the following priors in logit space:

$$\alpha _{i,j}^{sync} \sim\ ~Uniform\left( { - 4,4} \right)$$

$${\beta ^{sync}} \sim\ ~Normal\left( {0, sd = 1} \right)$$

### Shark movement

Leveraging information from the sync tag model described above, we fit a Bayesian state space model (SSM) designed to partition true nursery occupancy dynamics from recorded shark detection data. SSMs are a class of statistical models that estimate the state of an unobserved process from observed data [59]. These models are flexible and allow users to model variability in ecological processes separately from observation processes [60], which are expressed as distinct, but interactive sub-models within the SSM framework.

### State process

We modeled hourly presence or absence of individual sharks within nursery habitats (as defined by the extent of the receiver array) as a latent two-state process, where observed shark detections are conditional on true (unobserved) states of behavior. We let ${{\rm{{\rm Z}}}_{i,t}} \in \left\{ {0, 1} \right\}$ denote the true behavioral state (e.g., presence or absence) of shark *i* within the array at time *t*. Similarly, we let $y_{i,j,t}^{det} \in \left\{ {0, 1} \right\}$ represent the detection of shark *i* on receiver *j* at time *t*. We modeled ${{\rm{{\rm Z}}}_{i,t}}$ using a Bernoulli distribution that evolves forward in time as a first-order Markov process.

$$\begin{aligned}&{{\rm{{\rm Z}}}_{i,t }}| {{\rm{{\rm Z}}}_{i,t - 1 }}\ \sim~ Bernoulli\left( {{\psi _{i,t}}} \right),\cr& {\psi _{i, t}} = \left\{ {\matrix{ {1 - \phi _{i,t}^{emigration}\,if\,{{\rm{{\rm Z}}}_{i,t - 1 }} = 1 } \cr {{\phi ^{return}}\,\,\,\,\,\,\,\,\,\,\,\,\,\,\,\,\,\,\,\,if\,{{\rm{{\rm Z}}}_{i,t - 1 }} = 0 } \cr } } \right.\end{aligned}$$

Here, ${\psi _{i,t}}$ represents the probability of transitioning between states, where we parameterize two scenarios of interest. In this model formulation, we define $\phi _{i, t}^{emigration}$ as the probability of a shark leaving the nursery, given that it was present at the previous timestep. We also define ${\phi ^{return}}$ as the probability of a shark returning to the nursery, given that it was absent at the previous timestep.

We then used a logit link function to examine the relative time-varying effects of wind speed (WIND_t_), significant wave height (WAVES_t_), barometric pressure (BARO_t_), sea surface temperature (SST_t_), salinity (SAL_t_), and turbidity (TURB_t_) on the probability of shark emigration, $\phi _{i,t}^{emigration}$. To account for potential diurnal patterns of emigration, we included hour of day (HOUR_t_) as a cyclic effect using sine and cosine functions across a 24-hour period. Lastly, to account for heterogeneity in emigration probabilities across individual sharks, we included a shark-level random intercept, $\alpha _i^{emigration}$. This allowed each shark’s baseline emigration probability to vary around a shared population mean while borrowing strength across individuals.

$$\begin{aligned}&logit\left( {\phi _{i,t}^{emigration}} \right) \cr&= \alpha _i^{emigration} + {\beta _{\sin }}\sin \left( {{{2\pi HOU{R_t}} \over {24}}} \right) \cr&+ {\beta _{\cos }}\cos \left( {{{2\pi HOU{R_t}} \over {24}}} \right)+ {\beta _{wind}}WIN{D_t} \cr&+ {\beta _{waves}}WAVE{S_t} + {\beta _{baro}}BAR{O_t} \cr&+ {\beta _{sst}}SS{T_t} + {\beta _{sal}}SA{L_t} + {\beta _{turb}}TUR{B_t}\end{aligned}$$

$$logit\left( {{\phi ^{return}}} \right) = {\alpha ^{return}}$$

$$\begin{aligned}\alpha _i^{emigration} & \sim ~Normal\left( {{\mu _a}, \sigma _a^2} \right),\\&{\mu _a} \sim ~Normal\left( {0, sd = 2} \right),\\&\sigma _a^{ - 2} = {\tau _{a }} \sim ~Gamma\left( {1,1} \right)\\&{\alpha _{return}} \sim ~Normal\left( {0, sd = 2} \right)\\&\beta \sim ~Normal\left( {0, sd = 2} \right)\end{aligned}$$

Parameters $\alpha _i^{emigration}$ and ${\alpha ^{return}}$ represent intercept terms, while all $\beta$ terms represent slope coefficients. In contrast to emigration probabilities, return probabilities were assigned a fixed intercept (${\alpha ^{return}}$) and did not vary as a function of environmental predictors. This modeling decision arose from efforts to limit model complexity, as extending covariate effects to return probabilities risked introducing unstable or poorly identified parameter estimates.

### Observation process

We modeled observed detections of tagged sharks *i* on individual receivers *j* through time *t* as a Bernoulli process, with detections $y_{i,j,t}^{det}$ conditional on the true unobserved state variable ${Z_{i,t}}$. However, we also leveraged prior information gleaned from the sync tag model to estimate how observed shark detections may have been influenced by wind speed, significant wave height, ambient noise, receiver tilt, and animal transmitter density (e.g., the number of animal tags detected by the receiver array in a given hour).

$$y_{i,j,t}^{det} \left| { {Z_{i, t }}} \right.\sim ~Bernoulli\left( {{Z_{i, t }} \times p_{j,t}^{det}} \right)$$

$$\begin{aligned}logit\left( {p_{j,t}^{det}} \right)& = \alpha _j^{det} + \beta _{wind}^{det}WIN{D_t} \cr&\quad+ \beta _{waves}^{det}WAVE{S_t}+\beta _{noise}^{det}NOIS{E_t}\cr&\quad + \beta _{tilt}^{det}TIL{T_t} + \beta _{sharks}^{det}SHARK{S_t}\end{aligned}$$

Here, $\alpha _j^{det}$ represents the baseline log-odds of detection for each receiver *j* in the array, which arise from

$${\theta _j} \sim ~Beta\left( {1,1} \right), \alpha _j^{det} = logit\left( {{\theta _j} } \right)$$

Parameters ${\beta ^{det}}$ represent slope coefficients describing how the effect of wind, waves, ambient noise, receiver tilt, and transmitter density modify that baseline. For each ${\beta ^{det}}$ parameter, we assigned an *informative* normal prior.

$$\begin{aligned}&\beta _x^{det} \sim ~Normal\left( {\mu _x^{sync}, {{\left( {\sigma _x^{sync}} \right)}^2}} \right),\cr& x \in \left\{ {wind, waves, noise, tilt, sharks} \right\}\end{aligned}$$

where $\mu _x^{sync}$ and $\sigma _x^{sync}$ were set equal to the posterior mean and standard deviation of the corresponding slope coefficient estimated by the sync tag model. This enabled us to effectively share information and borrow strength across the two models and correct for potential reductions in receiver performance during the storm. To evaluate the influence of this correction on our results, we also fit a reduced version of the model that excluded covariate effects and assumed a constant detection probability through time that was specific to each receiver. This allowed us to assess the extent to which receiver performance adjustments influenced estimated emigration rates and covariate effects.

We performed all analyses using the statistical software R, version 4.5.0 [61]. We fit all models using the R package NIMBLE (Numerical Inference for statistical Models Using Bayesian and Likelihood Estimation) [62], which adopts and extends the BUGS (Bayesian inference Using Gibbs Sampling) modeling language. All covariates were standardized to a mean of zero and unit variance prior to model fitting. For each model, we ran three Markov chains concurrently with 10,000 iterations per chain. For each chain, we discarded the first 1,000 samples as burn-in and retained every 10^th^ iteration in efforts to reduce autocorrelation. This resulted in 900 samples of the posterior distribution per chain (2,700 total). We confirmed convergence of posterior parameter estimates through visual inspection of trace plots and calculated Gelman-Rubin (R-hat) scores [63]. A convergence threshold of R-hat < 1.05 was used to assess chain mixing. All parameters across all models satisfied this threshold criteria.

## Results

### Storm conditions

**Fig. 2:**
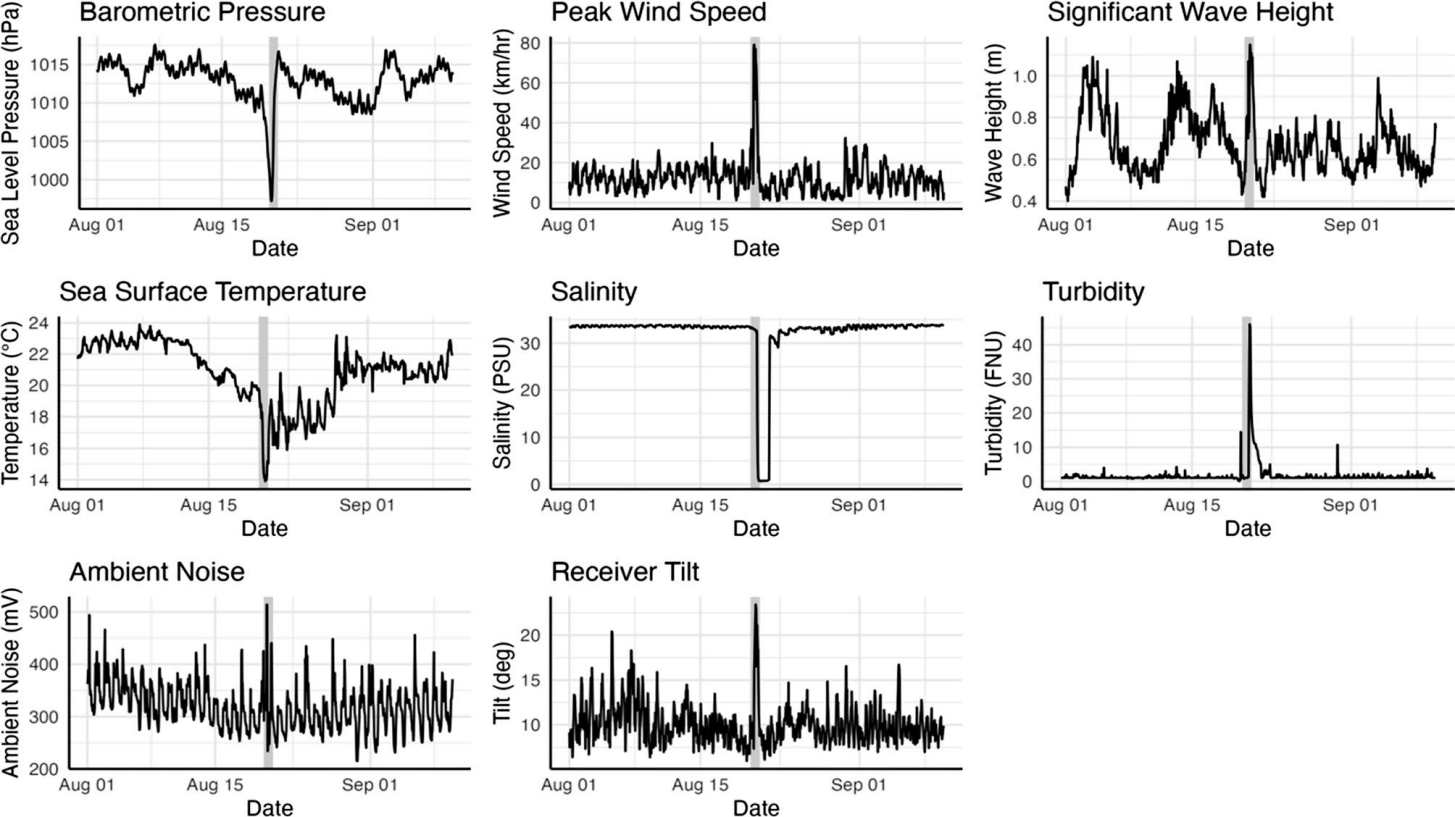
Hourly time series of environmental covariates and receiver diagnostic data from 01 August to 10 September 2023. Gray boxes indicate the duration of Tropical Storm Hilary in San Diego, CA

Tropical Storm Hilary brought brief, yet acute changes to local environmental conditions throughout our study site (Fig. 2). The storm’s arrival was preceded by a rapid drop in barometric pressure, followed by strong southerly gale-force winds approaching 80 km/hour. Observed increases to significant wave height were modest during the storm in nearshore habitats, as beaches along the coast of Torrey Pines and Del Mar are situated in the lee of Point La Jolla and therefore protected from southern winds (Fig. 1b). However, much larger swell was observed further offshore [64]. Nevertheless, storm-induced mixing throughout the region caused SST to drop rapidly by 6 °C in 12 hours, with cooler conditions persisting for approximately one week before gradually returning to pre-storm temperatures. Records of ambient noise and receiver tilt logged on VR2Tx receivers also peaked during the storm.

### Array performance

Between 01 August and 10 September 2023, our 40-receiver array detected 470,039 transmissions from 20 active sync tags. Using sync tag detections across 800 potential sync tag-receiver pairings, we quantified the relative effects of sync tag - receiver distance, receiver depth, wind speed, significant wave height, ambient noise, receiver tilt, and transmitter density on hourly sync tag detection probabilities (Additional File 1, Figure S1). Sync tag - receiver distance, as anticipated, had the most pronounced effect on detection efficiency, with rapid signal attenuation with increasing distance. Receiver depth also influenced sync tag detection probability, with deeper receivers, on average, displaying higher detection rates, likely due to reduced exposure to surface turbulence. Although static covariates emerged as the most influential predictors, wind speed, significant wave height, ambient noise, receiver tilt, and transmitter density all produced discernable secondary effects, as regression coefficients for these covariates were all exclusively negative (Figure S1). Of these, ambient noise, receiver tilt, and transmitter density emerged as particularly influential drivers of receiver detection efficiency.

**Fig. 3:**
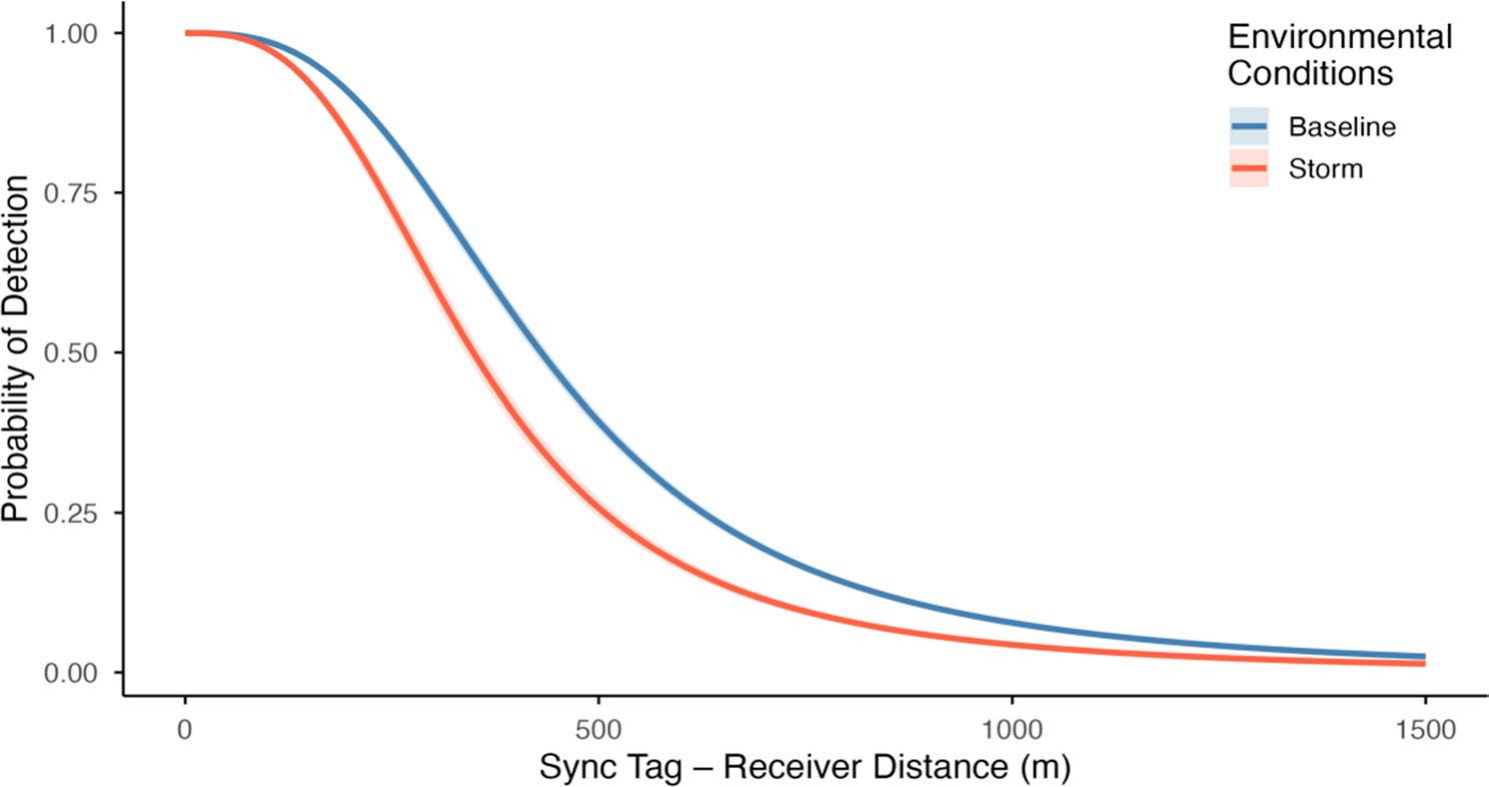
Counterfactual plot comparing predicted hourly detection probabilities over a range of sync tag - receiver distances for baseline conditions (blue) and storm conditions (red). Solid curves represent median predicted values while shaded regions indicate 95% credible intervals. We define storm conditions as all hours between 2023-08-20 08:00 and 2023-08-21 08:00, corresponding to peak disturbance periods

Figure 3 presents counterfactual predictions of hourly sync tag detection probabilities under storm versus non-storm conditions, evaluated across varying sync tag - receiver distances while holding receiver depth constant at the array-wide mean. At shorter distances (e.g., 100 m), storm impacts were negligible, with hourly detection probabilities averaging 0.986 [95% credible interval: 0.984, 0.988] for baseline conditions and 0.975 [0.971, 0.978] for storm conditions. However, at greater distances (e.g., 400 m), storm impacts were more pronounced, with hourly detection probabilities declining from 0.553 [0.538, 0.568] under baseline conditions to 0.400 [0.380, 0.420] during the storm.

### Shark movement

**Fig. 4:**
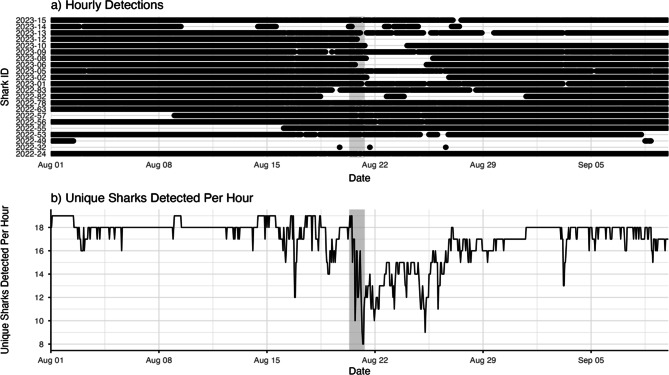
**a**) Hourly detections of tagged JWS detected in our array from 01 August to 10 September 2023. **b**) Number of unique sharks detected per hour. The gray box indicates the duration of Tropical Storm Hilary in San Diego

**Fig. 5:**
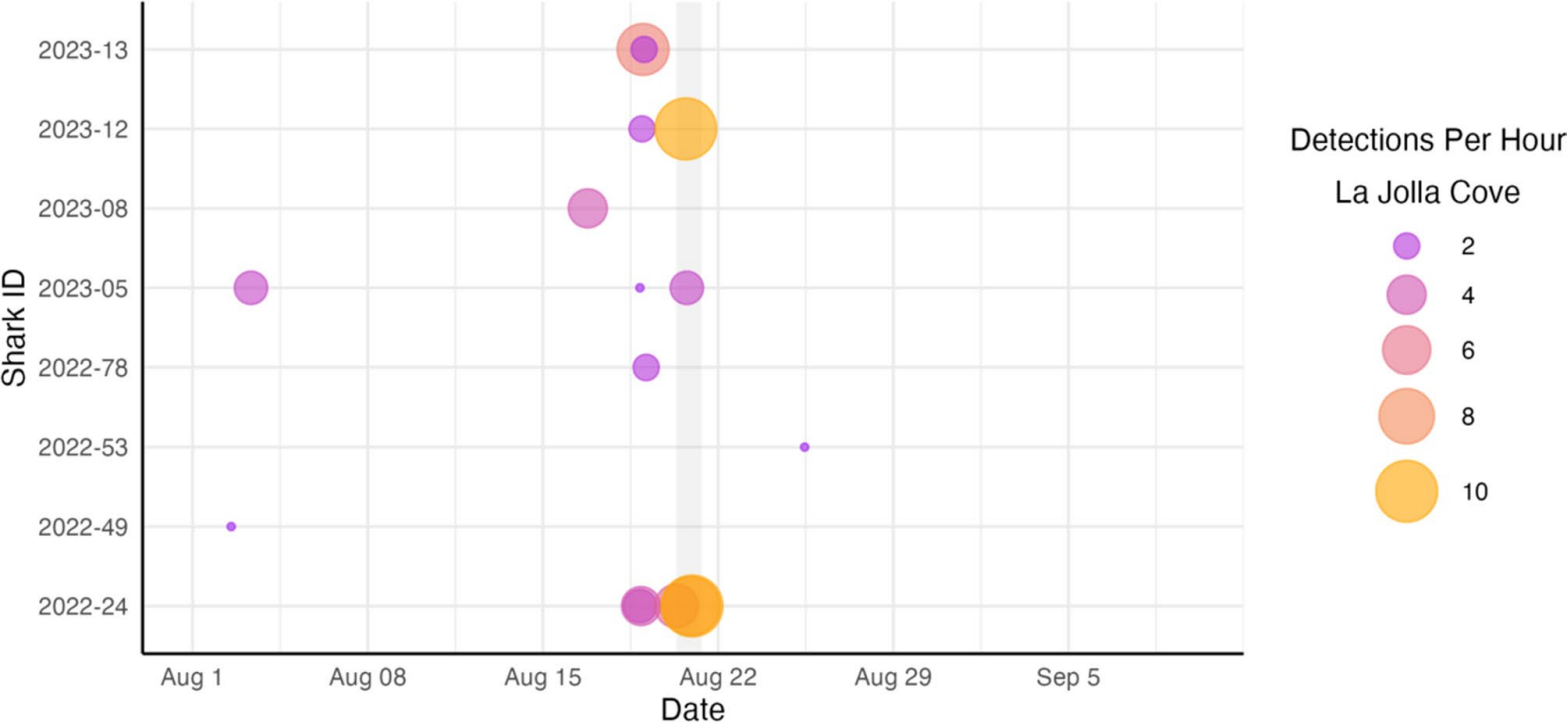
Abacus plot displaying recorded JWS detections on receivers in La Jolla Cove, located in a more sheltered area approximately 7 km south of the Torrey Pines aggregation site

Throughout our study period, we detected a total of 22 unique sharks (13 female, 9 male), all of which were tagged in the San Diego aggregation site (Table 2). This amounted to over 524,000 recorded shark detections. Prior to the storm’s arrival, we consistently detected 16–19 individuals per hour throughout the array (Fig. 4), with individuals displaying high levels of site fidelity to this nursery habitat. However, this number dropped to as few as 8 animals during the storm. Most emigrations were temporary, lasting from 2 hours to 15 days. Nearly all sharks eventually returned to the nursery within three weeks of the storm, except for one individual that left when the storm arrived and was never detected again by our fine-scale array in 2023. Prior to and during the storm, we also observed a discernable increase in JWS detections on receivers in the more sheltered La Jolla Cove (Fig. 5), an area where JWS were typically detected much less frequently.

**Fig. 6:**
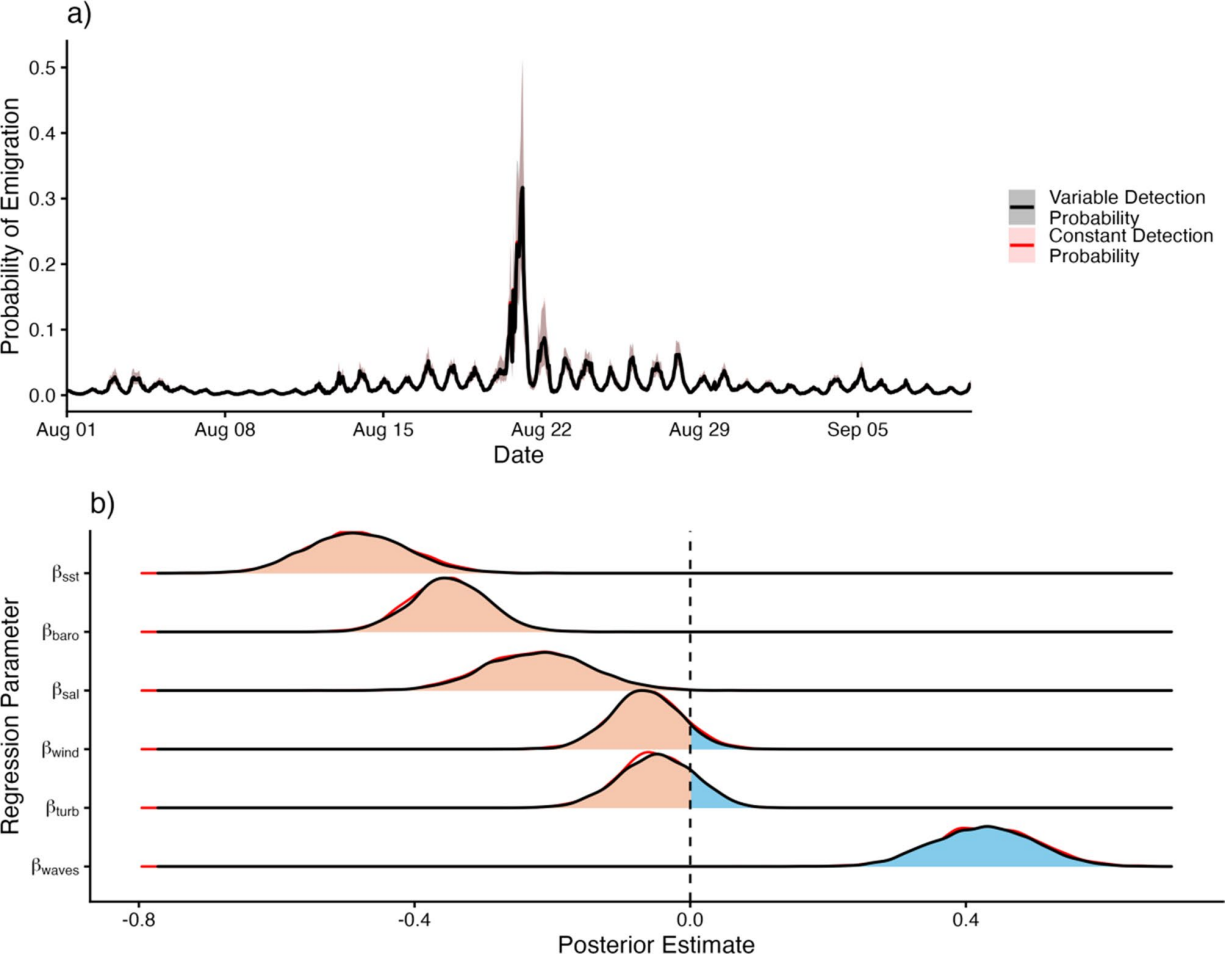
**a**) Model-predicted temporary emigration probabilities for the model accounting for variable receiver detection probabilities through time (black) and constant receiver detection probabilities (red). The gray box indicates the duration of Tropical Storm Hilary in San Diego, CA. **b**) Regression slope coefficients quantifying the effect of sea surface temperature (${\beta _{sst}}$), barometric pressure (${\beta _{baro}}$), wind speed (${\beta _{wind}}$), significant wave height (${\beta _{waves}}$), salinity (${\beta _{sal}}$), and turbidity (${\beta _{turb}}$) on JWS emigration probabilities. Estimated effects for the model assuming constant receiver detection probabilities are again shown in red

Using regression parameter outputs from our SSM, we predicted shark emigration probabilities for each hour in our study period (Fig. 6a). Across all unique sharks and time steps, median emigration probabilities were low (0.011 [95% credible interval: 0.002, 0.075]), with slightly higher emigration probabilities observed at night. However, during the storm, hourly emigration probabilities peaked at 0.311 [95% credible interval: 0.162, 0.510], representing more than a 28-fold increase above baseline values.

We report strong associations between JWS emigration probabilities and several environmental storm cues (Fig. 6b). Falling SST exerted the most pronounced effect, with the fewest sharks detected per hour during the storm directly aligning with the SST minimum. Increased wave action, falling barometric pressure, and drops in salinity produced discernable secondary effects, although these covariates exerted less influence than SST. Wind speed and turbidity were the least influential emigration predictors, as posterior slope estimates for both of these parameters spanned zero. The emigration rates and covariates effects we report here were nearly identical to those of the reduced model that did not account for temporal changes in detection probability (Fig. 6b). This suggests that although the performance of individual receivers may have been altered during storm conditions, the performance of the array as a whole in its ability to detect tagged animals on an hourly basis was not impacted.

**Fig. 7:**
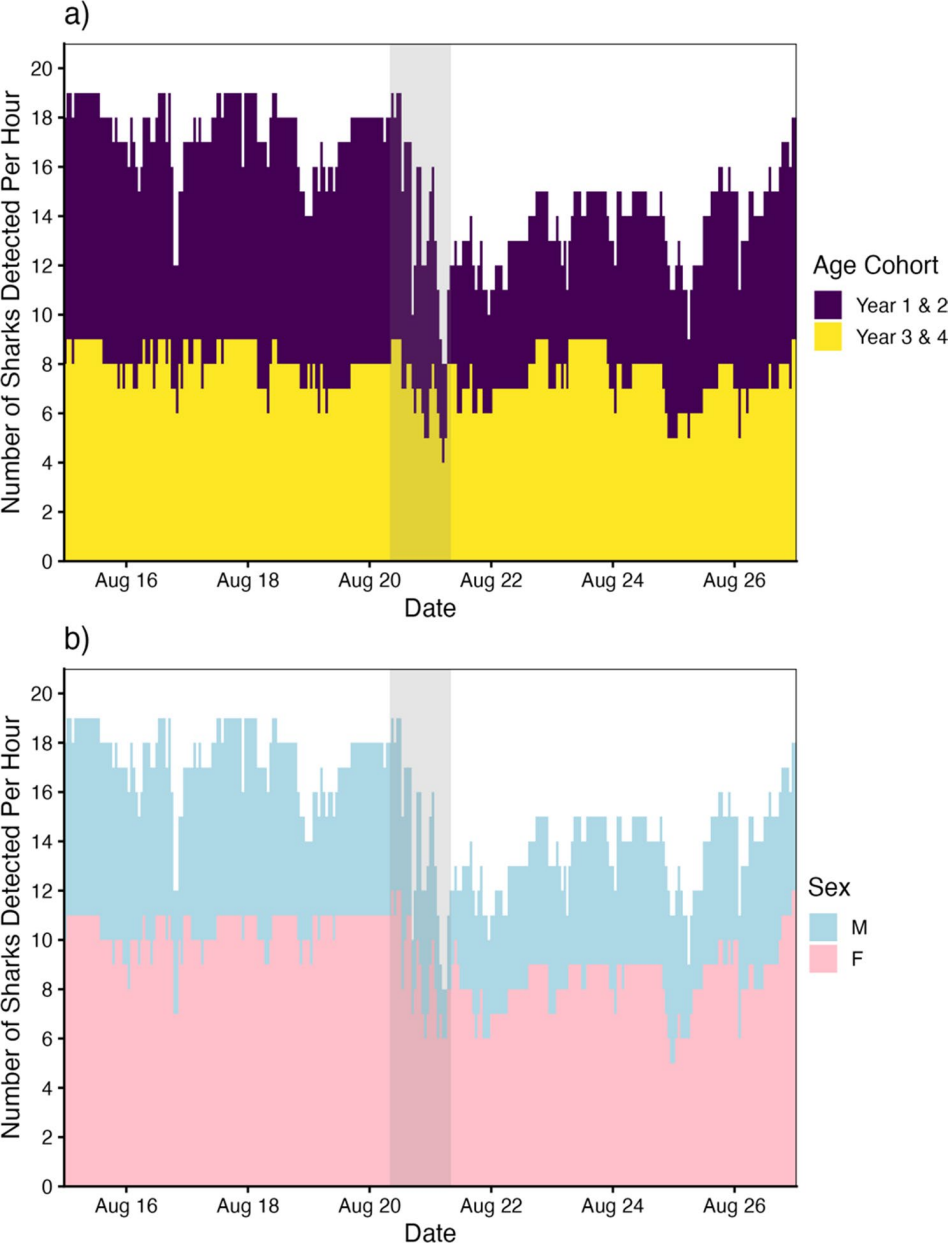
Number of unique JWS detected per hour, separated by **a**) shark age cohort and **b**) sex

Lastly, we also observed notable differences in storm response behavior across both shark year cohorts and sex (Fig. 7). Von Bertalanffy-derived estimates of shark age at the time of the storm ranged from 1.3 - 4.3 years. While emigration behavior was observed across all age classes, younger sharks (years 1–2) appeared to display higher degrees of nursery emigration during the storm than their older conspecifics (years 3–4) (Fig. 7a). Similarly, male sharks showed a greater propensity to emigrate during the storm than females, although this patten is likely driven by the overrepresentation of males in younger age cohorts (Fig. 7b).

## Discussion

In Southern California, JWS typically occupy coastal aggregation sites in the summer and fall months [14, 45], when nearshore ocean temperatures are relatively warm and seas are calm. These environmental conditions are thought to allow for rapid growth and enhanced juvenile survivorship [45, 65, 66], while high prey densities in nearshore ecosystems maximize foraging opportunities [67]. The arrival of Tropical Storm Hilary represented a marked departure from seasonal norms, bringing about high winds, record rainfall, rapid drops in sea surface temperature, and abrupt changes to nearshore biogeochemistry. Although Tropical Storm Hilary’s impact on the San Diego region was less severe than anticipated, these environmental shifts provided a unique opportunity to understand how JWS modify baseline nursery behaviors to meet survival needs in the face of storm disturbance.

### Array performance

Prior to examining JWS movement behaviors during Tropical Storm Hilary, it was first necessary to disentangle potential biological responses from storm-driven shifts in array performance. Environmental factors such as wind, waves, and ambient noise are well-established controls of acoustic receiver performance [34–38, 40, 57, 58, 68, 69] and have been shown to bias biological inference when not carefully considered [39]. However, no standard protocol currently exists to quantify and correct for this type of observation error in acoustic telemetry studies [70]. To address this challenge, we leveraged detection data from sync tags, which are standard components of many acoustic receiver arrays, to quantify how key storm covariates influenced transmitter detection probability. Our analysis revealed ambient noise, receiver tilt, and transmitter density as the dominant temporally variable drivers of individual receiver performance, with wind speed and wave height producing discernable secondary effects. Incorporating these estimated effects into the observational component of our SSM allowed us to probabilistically separate tagged shark presence/absence from storm-induced observational artifacts. Although developed for our system, we believe this approach to be tractable and useful for comparable studies in dynamic, high-energy aquatic environments.

Although the performance of individual receivers may have been reduced during the storm, we ultimately determined that the storm had only minimal impact on the performance of the array as a whole in its ability to provide information on JWS nursery occupancy. Overlapping receiver detection ranges and multiple transmissions per hour provided high levels of observational redundancy within our network, so that even if individual receivers failed to detect individual transmissions, it is unlikely that a shark present within the array would go undetected by all receivers within an hourly interval.

### Temporary nursery evacuation

We tested the hypothesis that JWS would, at least temporarily, evacuate from nearshore habitats in response to acute storm-related stressors. Top marine predators such as white sharks are theoretically well-adapted to respond to episodic weather and climate extremes, given their sensory capabilities to detect environmental changes and high levels of mobility. These attributes allow individuals to identify deteriorating environmental conditions, avoid habitats they find unsuitable, and learn from past experiences to minimize risk and negative energetic impacts [15, 71]. Our findings provide strong support for this hypothesis. Prior to the arrival of Tropical Storm Hilary on 20 August 2023, sharks exhibited high degrees of site fidelity and spatially-restricted movements within their aggregation site (Fig. 4a). These observations are consistent with other studies conducted at other JWS nursery aggregations throughout the region [42–45, 47]. However, with the onset of storm conditions, many sharks temporarily evacuated from nearshore habitats, a decision likely driven by the need to minimize exposure to acute storm stressors. Yet, these emigrations were short-lived, with all but one shark returning to the aggregation site as calm weather returned in the days to weeks that followed.

In most cases, we cannot determine exactly where sharks that left the nursery habitat went, although we originally hypothesized that individuals would evacuate to deeper habitats further offshore where the effects of storm-induced turbulence would be less severe. However, just prior to the storm’s arrival and during the storm itself, we observed a marked increase in JWS detections in La Jolla Cove (Fig. 5). We believe this more sheltered habitat may have served as an important nearshore refuge area for some individuals. In addition to being protected from strong southern winds during the storm, nearshore habitats in La Jolla Cove are situated directly adjacent to a prominent submarine canyon with access to deep water exceeding 200 meters [72]. On the day of the storm, one shark equipped with a pressure sensor was detected in La Jolla Cove at a depth of 132 m, clearly indicating that it was in La Jolla Canyon, yet still within detection range of the receiver. Although perhaps anecdotal, this represented the deepest dive recorded in our monitoring period and may reflect an individual’s attempt to escape turbulent conditions at the surface. It remains possible, therefore, that canyon habitats may function as critical refugia [73], which has been demonstrated for other local species such as rockfish [74]. In the present study, nearshore habitats in La Jolla Canyon may have provided individuals with critical protection from storm stressors without requiring extensive, energetically costly offshore movements.

### Evacuation cues

Our results suggest that JWS nursery evacuations were initiated by several distinct environmental cues. Within our SSM framework, we examined the influence of key storm predictors on behavioral state transition probabilities, with a particular focus on understanding factors that influenced nursery emigration. During peak storm conditions, shark emigration probabilities increased by a factor of 28, with falling sea surface temperatures emerging as the most influential predictor of emigration. As a species, white sharks exhibit regional endothermy, allowing individuals to maintain internal body temperatures above ambient conditions [75, 76]. This enables white sharks to inhabit colder waters while still being able to hunt agile, highly mobile endothermic prey [76]. However, juvenile white sharks are more sensitive to colder temperatures than adult conspecifics, as endothermic efficiency likely scales with body size [42]. To compensate for greater thermal sensitivity, JWS may rely on behavioral thermoregulation, whereby individuals actively seek out habitats within their thermal preferenda and avoid or emigrate from habitats below thermal tolerance thresholds.

The temperature relationships we report here strongly corroborate the findings of Spurgeon et al., 2022, which documented nearly complete JWS nursery emigration in response to a strong coastal upwelling event, which also caused an abrupt drop in nearshore SST. Within this study, sharks initiated searches for warmer water within 10–12 hr of the upwelling event. In addition, most emigrations occurred only after sea surface temperatures dropped below 14 °C, which has been identified as an important thermal tolerance threshold for JWS [14, 43, 44]. In our study, nursery emigrations also closely mirrored falling sea surface temperatures during Tropical Storm Hilary, which dropped from 20 °C to 13.9 °C in less than 24 hrs. However, in contrast to Spurgeon et al., 2022, where prolonged periods of SST below 14 °C caused most sharks to emigrate for the season, we observed only a brief (1–4 hour) dip below 14 °C, with a return of nearly all sharks to the aggregation site as temperatures rebounded in the following weeks. These findings highlight the behavioral plasticity of JWS with varying temperature cues, as well as the ability of JWS aggregations to rapidly reform following acute thermal disturbance.

We also identified falling barometric pressure as a potential secondary evacuation cue. Barometric pressure is commonly cited as a driver of animal movement in marine systems and has been demonstrated to trigger emigrations from coastal or estuarine habitats in advance of approaching storms. Heupel et al., 2003 documented a complete emigration of acoustically tagged juvenile blacktip sharks (*Carcharhinus limbatus*) from a Florida estuary hours before Tropical Storm Gabrielle made landfall. Similarly, Strickland et al., 2020 described emigration of juvenile bull sharks from the Florida Everglades just prior to the arrival of Hurricane Irma. In our study, nearly all emigrations came after the arrival of Tropical Storm Hilary, indicating that movements out of nearshore habitats occurred in response to peak storm conditions rather than in anticipation of them. Nevertheless, strong associations between barometric pressure and shark emigration probabilities (Fig. 6b) suggest that drops in barometric pressure may have acted as an additional flight cue.

Changes to local biogeochemical conditions may have also prompted nursery evacuations. Our results indicate that JWS emigration probabilities were well-correlated with drops in salinity (Fig. 6b), which aligns with expectations for a fully marine species that is physiologically adapted to ocean salinity conditions. During Tropical Storm Hilary, heavy precipitation, runoff, and riverine flooding drastically increased rates of freshwater input to nearshore ecosystems [41], particularly at the mouth of estuarine outflows [77]. Salinity measurements recorded at the mouth of the Los Peñasquitos Lagoon, which drains directly into our study site, dropped from ~33.5 psu to as low as 0.7 psu on the day of the storm (Fig. 2). Although these values were taken within the narrow estuary channel and are not fully representative of salinity regimes in coastal habitats, they effectively captured the timing and magnitude of the freshwater plume that coincided with JWS evacuations. In contrast with salinity, JWS did not appear to respond to observed turbidity spikes (Fig. 2), although increased turbidity levels have been shown to limit foraging opportunities and reduce capture rates of prey in other systems [78–80].

Understanding the effects of wind speed and wave height on shark movement during the storm requires slightly more nuance. However, during this particular storm event, we consider these covariates to be the least influential in terms of their direct effects. Further offshore, strong southern winds generated a brief, yet considerable build-up of swell throughout the Southern California Bight [64]. However, because our study site was situated in the lee of Point La Jolla, most wave energy from the south was blocked, maintaining wave heights that were not unusual for this site (Fig. 2). For this reason, while swell remained a significant driver of JWS emigration across the broader study period, its influence during Tropical Storm Hilary was almost certainly muted. Wind speed showed an even weaker association with JWS emigration probabilities, as posterior effect size estimates spanned zero and indicated little predictive value on a local scale. However, on a regional scale, both wind and waves mediated JWS movement behaviors indirectly through temperature, as storm-driven mixing destratified the water column and ultimately led to regional ocean cooling.

### Partial evacuation & nursery return

Despite the patterns of evacuation we observed, not all sharks left the aggregation site during the storm, highlighting potential intraspecific differences in storm response behavior. Although reasons for this incomplete evacuation are unknown and beyond the scope of this study, such variability may possibly reflect ontogenetic differences in physiology and individual experience [43, 47]. Although we documented storm-related evacuations across all age cohorts, responses were most pronounced among younger sharks (Fig. 7a). This may be the result of size-related differences in thermal tolerance [42], where smaller, more temperature-sensitive individuals were forced to emigrate and larger, more cold-resistant individuals remained. Alternatively, larger, older individuals may have experienced episodic habitat disturbances before and, thus, be more willing to remain in a known place with adequate resources. Still others may have remained in the aggregation site to take advantage of new foraging opportunities brought about by the storm. Regardless, these findings highlight the increased vulnerability of younger JWS to environmental disturbance, which may heighten conservations concerns in a changing climate.

The return of nearly all JWS to the aggregation site after the storm represents another key finding. Our observations demonstrate that many sharks temporarily vacated nearshore nursery habitats, likely in response to anticipated or realized reductions in habitat quality. Once environmental conditions stabilized, however, the aggregation reformed in the exact same location, which has been observed in other shark nurseries [9, 65, 71]. This highlights the ecological importance of this particular habitat relative to others nearby [65], as well as the ability of JWS aggregations to recover from environmental disturbance, provided that key habitats are not permanently degraded.

## Conclusion

Extreme weather events pose significant threats to coastal ecosystems worldwide due to their rapid onset, severity, and unpredictable nature [4, 5, 7, 9, 18]. These challenges are further compounded in many contexts by a limited understanding of the vulnerability and resilience of species being impacted [71]. Although tropical storms are extremely rare in Southern California, they are projected to become more frequent as climate change intensifies [81, 82]. Global poleward shifts in tropical cyclone activity [83, 84], when coupled with limited historical exposure to such disturbances [85], may leave Southern California ecosystems particularly vulnerable when these disturbances do occur. Therefore, building capacity to monitor, understand, and predict storm impacts represents an emerging conservation and management priority for the region’s coastal species.

In this study, we examined the behavioral responses of JWS to the first documented tropical storm to impact Southern California in the 21^st^ century. To our knowledge, our efforts represent the first to describe storm-response behavior in white sharks, providing novel insights into how extreme weather events may impact the spatial ecology and nursery behaviors of a recovering top predator. We observed clear patterns of nursery evacuation in response to peak storm conditions, followed by rapid aggregation reformation as baseline conditions returned. Evacuations were most closely aligned with falling sea surface temperatures, although drops in barometric pressure, increased wave action, and drops in salinity may have served as important secondary flight cues. Together, these findings highlight the vulnerability and adaptive capacity of JWS to environmental disturbance, thus providing vital information for predicting and managing the ecological consequences of weather and climate extremes.

## Electronic supplementary material

Below is the link to the electronic supplementary material.

Supplementary Material 1

## Data Availability

All data associated with this project is available at: https://figshare.com/articles/dataset/JWS-Storm-Response-Manuscript-Data/30403114.

## Abbreviations

JWS: Juvenile White Sharks
VPS: Vemco Positioning System
TDOA: Time Difference of Arrival
MCMC: Markov Chain Monte Carlo
SSM: State Space Model
SST: Sea Surface Temperature
CI: Credible Interval
