# Supplementary material for: A rare tropical storm event drives partial nursery evacuation by juvenile white sharks, followed by rapid aggregation reformation
Source: Mov Ecol. 2026 Mar 4;14:27. doi: 10.1186/s40462-026-00642-0 (PMC13094277; doi:10.1186/s40462-026-00642-0)
Supplement: Supplementary file 1 — Supplementary Material 1 [file 40462_2026_642_MOESM1_ESM.docx]

**Additional File 1**


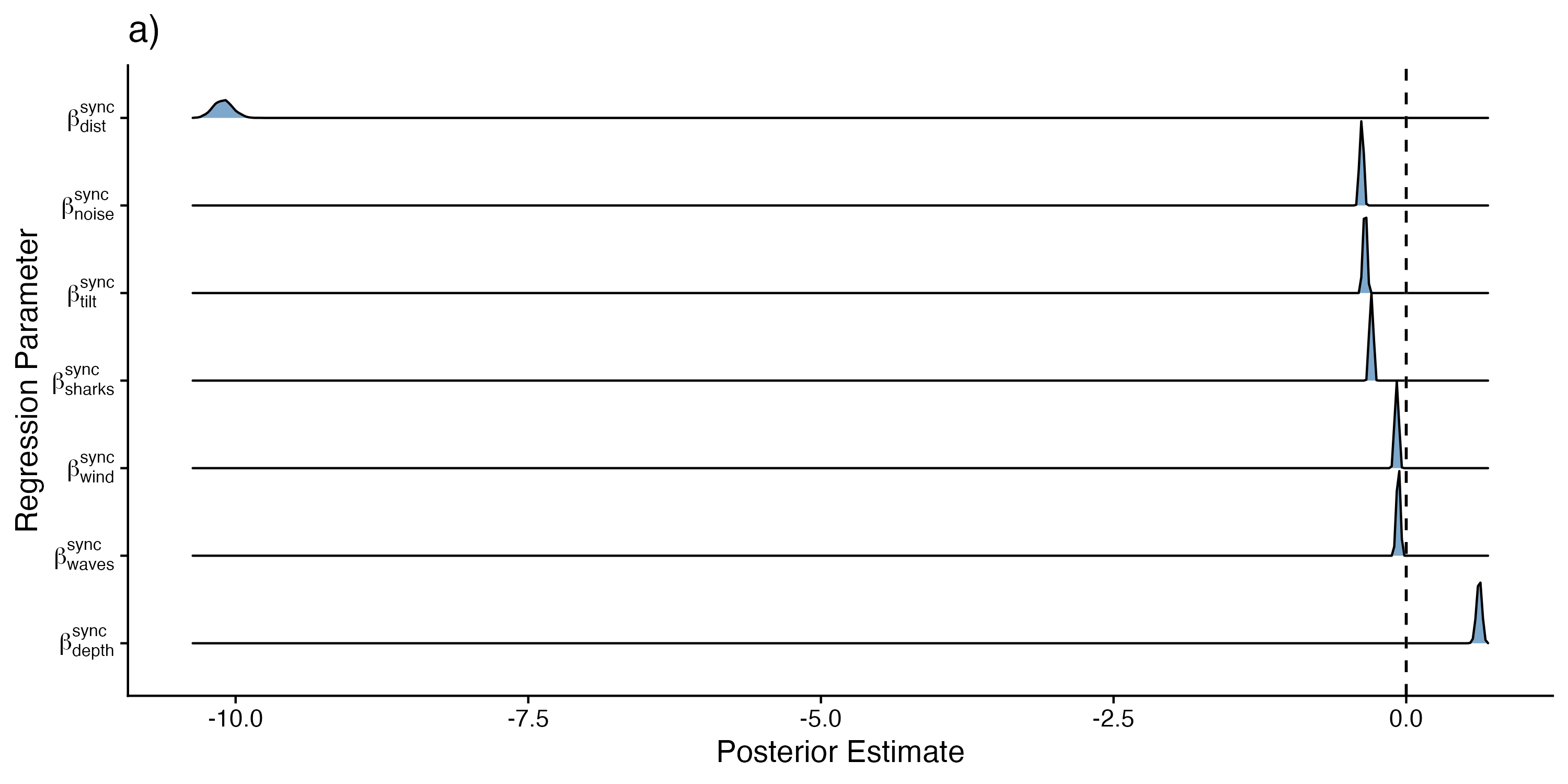


**Figure S1.** Regression slope coefficients quantifying the effect of sync tag-receiver distance ($\beta_{dist}^{sync}$), receiver depth ($\beta_{depth}^{sync}$), wind speed ($\beta_{wind}^{sync}$), significant wave height ($\beta_{waves}^{sync}$), ambient noise ($\beta_{noise}^{sync}$), receiver tilt ($\beta_{tilt}^{sync}$), and animal transmitter density ($\beta_{sharks}^{sync}$) on sync tag detection probabilities. Note that receiver depth measurements are positive downward.
